# Kernel Methods for Nonlinear Connectivity Detection

**DOI:** 10.3390/e21060610

**Published:** 2019-06-20

**Authors:** Lucas Massaroppe, Luiz A. Baccalá

**Affiliations:** 1 Instituto de Astronomia, Geofísica e Ciências Atmosféricas, Department of Atmospheric Sciences, University of São Paulo, São Paulo 05508-090, Brazil; 2 Escola Politécnica, Department of Telecommunications and Control Engineering, University of São Paulo, São Paulo 05508-900, Brazil

**Keywords:** nonlinear time series, nonlinear-Granger causality, inference

## Abstract

In this paper, we show that the presence of nonlinear coupling between time series may be detected using kernel feature space F representations while dispensing with the need to go back to solve the *pre-image problem* to gauge model adequacy. This is done by showing that the kernelized auto/cross sequences in F can be computed from the model rather than from prediction residuals in the original data space X. Furthermore, this allows for reducing the connectivity inference problem to that of fitting a consistent linear model in F that works even in the case of nonlinear interactions in the X-space which ordinary linear models may fail to capture. We further illustrate the fact that the resulting F-space parameter asymptotics provide reliable means of space model diagnostics in this space, and provide straightforward Granger connectivity inference tools even for relatively short time series records as opposed to other kernel based methods available in the literature.

## 1. Introduction

Describing ‘connectivity’ has become of paramount interest in many areas of investigation that involve interacting systems. Physiology, climatology, and economics are three good examples where dynamical evolution modelling is often hindered as system manipulation may be difficult or unethical. Consequently, interaction inference is frequently constrained to using time observations alone.

A number of investigation approaches have been put forward [1,2,3,4,5]. However, the most popular and traditional one still is the nonparametric computation of cross-correlation (CC) between pairs of time series, and variants thereof, like coherence analysis [6], even despite their many shortcomings [7].

When it comes to connectivity analysis, recent times have seen the rise of Granger Causality (GC) as a unifying concept. This is mostly due to GC’s unreciprocal character [8] (as opposed to CC) which allows for establishing the direction of information flow between component subsystems.

Most GC approaches rest on fitting parametric models to time series data and, again as opposed to CC, under appropriate conceptualization, also holds for more than just pairs of time series, giving rise to the ideas of (a) Granger connectivity and (b) Granger influentiability [9].

GC inferential methodology is dominated by the use of *linear* multivariate time series models [10]. This is so because linear models have statistical properties (and shortcomings) that are well understood besides having the advantage of sufficing when the data are Gaussian. As an added advantage GC characterization allows immediate frequency domain connectivity characterization via concepts like ‘directed coherence’ (DC) and ‘partial directed coherence’ (PDC) [11].

It is often the case, however that data Gaussianity does not hold. Whereas nonparametric approaches do exist [1,4,5], parametric nonlinear modelling offers little relief from the need for long observation data sets for reliable estimation in sharp contrast to linear models that perform well under the typical scenario of fairly short datasets over which natural phenomena can be considered stable. A case in point is neural data where animal behaviour changes are associated with relatively short-lived episodic signal modifications.

The motivation for the present development is that reproducing kernel transformations applied to data, as in the support vector machine learning classification case [12], can effectively produce estimates that inherit many of the good convergence properties of linear methods. Because these properties carry over under proper kernelization, it is possible to show that nonlinear links between subsystems can be rigorously detected.

Before proceeding, it is important to have in mind that the present developments focus on addressing the connectivity detection issue exclusively, in which we clearly show that solving the so-called *pre-image* reconstruction problem is unnecessary as has been until now assumed essential. This leads to a considerably simpler approach.

In Section 2, we formulate the problem and review some background about reproducing kernel theory together with the main results which are backed up by extensive numerical Monte Carlo illustrations in Section 3. Conclusions and current problem status and challenges end the paper (Section 4).

## 2. Problem Formulation

The most popular approach to investigating GC connectivity is through modeling multivariate time series via linear vector autoregressive models [10], where the central idea is to compare prediction effectiveness for a time series $x_{i}\left(n\right)$ when the past of other time series is taken into account in addition to its own past. Namely,

(1) $$x\left(n\right)=\sum_{k=1}^{p}A_{k}x\left(n-k\right)+w\left(n\right).$$

Under mild conditions, Equation (1) constitutes a valid representation of a linear stationary stochastic process where the evolution of $x\left(n\right)={\left[x_{1}\left(n\right),\cdots ,x_{D}\left(n\right)\right]}^{⊤}$ is obtained by filtering suitable $w\left(n\right)={\left[w_{1}\left(n\right),\cdots ,w_{D}\left(n\right)\right]}^{⊤}$ *purely stochastic innovation* processes, i.e., where $w_{i}\left(n\right)$ and $w_{j}\left(m\right)$ are independent provided n≠m [13]. If $w_{i}\left(n\right)$ are jointly Gaussian, so are $x_{i}\left(n\right)$ and the problem of characterizing connectivity reduces to well known procedures to estimate the $A_{k}$ parameters in Equation (1) via least squares, which is the applicable maximum likelihood procedure. Nongaussian $w_{i}\left(n\right)$ translate into nongaussian $x_{i}\left(n\right)$ even if some actual (1) linear generation mechanism holds. Linearity among nongaussian $x_{i}\left(n\right)$ time series may be tested with help of cross-polyspectra [14,15], which, if unrejected, still allows for a representation like (1) whose optimal estimation requires a suitable likelihood function to accommodate the observed non-Gaussianity.

If linearity is rejected, $x_{i}\left(n\right)$ non-Gaussianity is a sign of nonlinear mechanisms of generation modelled by

(2) $$x\left(n\right)=g(x\left(n_{-}\right),w\left(n\right)),$$

which generalizes (1) where $x(n_{-})$ stands for x(n)’s past under some suitable dynamical law g(·).

The distinction between (a) nonlinear $x_{i}\left(n\right)$ that are nonetheless linearly coupled as in (1) under nongaussian w(n) and (b) fully nonlinearly coupled processes is often overlooked. In the former case, linear methods suffice for connectivity detection [16] but fail in the latter case [17] calling for the adoption of alternative approaches. In some cases, however, linear approximations are inadequate in so far as to preclude connectivity detection [17].

In the present context, the solution to the connectivity problem entails a suitable data driven approximation of g(·) whilst singling out the $x_{i}\left(n\right)$ and $x_{j}\left(n\right)$ of interest. To do so, we examine the employment of *kernel* methods [18] where functional characterization is carried out with the help of a high dimensional space representation

(3) ϕ:X→F,

for F=dim(F)≫D=dim(X), where ϕ(x(n)) is a mapping from the input space X into the feature space F whose role is to properly unwrap the data and yet ensure that the inner product 〈ϕ(x)|ϕ(y)〉 can be written as a simple function of x and y dispensing with the need for computations in F. This possibility is granted by chosing ϕ(x) to satisfy the so-called *Mercer condition* [19].

A simple example of (3) is the mapping

(4) $$\phi :x\mapsto \langle \phi \left(x\right)|\;=\;{\left[c,\sqrt{2c}x,x^{2}\right]}^{⊤},$$

for x∈R and 〈ϕ(x)|∈F using Dirac’s bra-ket notation. In this case, the Mercer kernel is given by

(5) $$\kappa \left(x,y\right)=\langle \phi \left(x\right)|\phi \left(y\right)\rangle ={\left(c+xy\right)}^{2},$$

which is the simplest example of a polynomial kernel [18].

In the multivariate time series case, we consider

(6) $$\phi :x\left(n\right)\mapsto [\langle \phi_{1}\left(x_{1}\left(n\right)\right)|,\cdots ,\langle \phi_{i}\left(x_{i}\left(n\right)\right)|,\cdots ,{\langle \phi_{D}\left(x_{D}\left(n\right)\right)|]}^{⊤},$$

where, for simplicity, we adopt the same transformation $\phi \left(\cdot \right)=\phi_{i}\left(\cdot \right)=\phi_{j}\left(\cdot \right)$ for each $x_{i}\left(n\right)\in R$ time series component so that the

(7) 〈ϕ(x(n))|ϕ(x(m))〉=K(x(n),x(m))

is a matrix whose elements are given by $K_{ij}\left(n,m\right)=\langle \phi \left(x_{i}\left(n\right)\right)|\phi \left(x_{j}\left(m\right)\right)\rangle$. In the development below, we follow the standard practice of denoting the K(x(n),x(m)) quantities as K(m−n) in view of the assumed stationarity of the processes under study.

Rather than go straight into the statement of the general theory, a simple example is more enlightening. In this sense, consider a bivariate stationary time series

(8) $$\begin{array}{ccc} x_{1}\left(n\right) & = & g_{1}\left(x_{1}\left(n-1\right),w_{1}\left(n\right)\right), \end{array}$$

(9) $$\begin{array}{ccc} x_{2}\left(n\right) & = & g_{2}\left(x_{1}\left(n-1\right),x_{2}\left(n-1\right),w_{2}\left(n\right)\right), \end{array}$$

where $g_{i}\left(\cdot \right)$ are nonlinear functions and only the previous instant is relevant in producing the present behaviour. An additional feature, thru (9), is that $x_{1}\left(n\right)$ is connected to (Granger causes) $x_{2}\left(n\right)$ but not conversely. Application of the kernel transformation leads to

(10) $$\begin{array}{ccc} \langle \phi \left(x_{1}\left(n\right)\right)| & = & \langle \phi \left(g_{1}\left(x_{1}\left(n-1\right),w_{1}\left(n\right)\right)\right)|, \end{array}$$

(11) $$\begin{array}{ccc} \langle \phi \left(x_{2}\left(n\right)\right)| & = & \langle \phi \left(g_{2}\left(x_{1}\left(n-1\right),x_{2}\left(n-1\right),w_{2}\left(n\right)\right)\right)|. \end{array}$$

However, if one assumes the possibility of a linear approximation in F, one may write

(12) $$\left[\begin{array}{c} \langle \phi \left(x_{1}\left(n\right)\right)|\\ \langle \phi \left(x_{2}\left(n\right)\right)| \end{array}\right]=\left[\begin{array}{cc} \alpha_{11} & \alpha_{12}\\ \alpha_{21} & \alpha_{22} \end{array}\right]\left[\begin{array}{c} \langle \phi \left(x_{1}\left(n-1\right)\right)|\\ \langle \phi \left(x_{2}\left(n-1\right)\right)| \end{array}\right]+\left[\begin{array}{c} \langle {\tilde{w}}_{1}\left(n\right)|\\ \langle {\tilde{w}}_{2}\left(n\right)| \end{array}\right],$$

where $[\langle {\tilde{w}}_{1}\left(n\right)|\;{\langle {\tilde{w}}_{2}\left(n\right)|]}^{⊤}$ stands for approximation errors in the form of innovations. Mercer kernel theory allows for taking the external product with respect to $[|\phi \left(x_{1}\left(n-1\right)\right)\rangle \;|\phi \left(x_{2}\left(n-1\right)\right){\rangle ]}^{⊤}$ on both sides of (12) leading to

(13) K(x(n),x(n−1))=AK(x(n−1),x(n−1)),

after taking expectations on both sides where

(14) $$A=\left[\begin{array}{cc} \alpha_{11} & \alpha_{12}\\ \alpha_{21} & \alpha_{22} \end{array}\right]$$

and

(15) $$K\left(x\left(n\right),x\left(m\right)\right)=\left[\begin{array}{cc} E[\langle \phi \left(x_{1}\left(n\right)\right)|\phi \left(x_{1}\left(m\right)\right)\rangle ] & E[\langle \phi \left(x_{1}\left(n\right)\right)|\phi \left(x_{2}\left(m\right)\right)\rangle ]\\ E[\langle \phi \left(x_{2}\left(n\right)\right)|\phi \left(x_{1}\left(m\right)\right)\rangle ] & E[\langle \phi \left(x_{2}\left(n\right)\right)|\phi \left(x_{2}\left(m\right)\right)\rangle ] \end{array}\right],$$

since $E[\langle {\tilde{w}}_{i}\left(n\right)|\phi \left(x_{j}\left(m\right)\right)\rangle ]=0$ for n>m given that $\langle {\tilde{w}}_{i}\left(n\right)|$ plays a zero mean innovations role.

It is easy to obtain A from sample kernel estimates. Furthermore, it is clear that (8) holds if and only if $\alpha_{12}=0$.

To (13), which plays the role of Yule–Walker equations and which can be written more simply as

(16) K(−1)=AK(0),

and one may add the following equation to compute the innovations covariance

(17) $$\Sigma_{\langle \tilde{w}\left(n\right)|}=K\left(x\left(n\right),x\left(n\right)\right)-AK\left(x\left(n\right),x\left(n\right)\right)A^{⊤},$$

where only reference to the m−n difference is explicitly denoted assuming signal stationarity so that (17) simplifies to

(18) $$\Sigma_{\langle \tilde{w}\left(n\right)|}=K\left(0\right)-AK\left(0\right)A^{⊤}=K\left(0\right)-K\left(-1\right)A^{⊤}.$$

This formulation is easy to generalize to model orders p>1 and to more time series via

(19) $$\langle \phi \left(x\left(n\right)\right)|=\sum_{k=1}^{p}A_{k}\langle \phi \left(x\left(n-k\right)\right)|+\langle \tilde{w}\left(n\right)|,$$

where

(20) $$\langle \phi \left(x\left(n\right)\right)|=[\langle \phi \left(x_{1}\left(n\right)\right)|,\cdots ,{\langle \phi \left(x_{D}\left(n\right)\right)|]}^{⊤},$$

which is assumed as due to filtering appropriately modelled innovations $\langle \tilde{w}\left(n\right)|$. For the present formulation, one must also consider the associated ‘ket’-vector

(21) $$|\phi \left(x\left(m\right)\right)\rangle =[|\phi \left(x_{1}\left(m\right)\right)\rangle ,\cdots ,|\phi \left(x_{D}\left(m\right)\right){\rangle ]}^{⊤},$$

that when applied to (19) for n>m after taking expectations E[·] under the zero mean innovations nature of $\langle w^{\phi }\left(n\right)|$ leads to

(22) $$K_{x}^{\phi }\left(l\right)=\sum_{k=1}^{p}A_{k}K_{x}^{\phi }\left(l+k\right),$$

where l=m−n and $K_{x}^{\phi }\left(m\right)$’s elements are given by $E[\langle \phi \left(x_{i}\left(l-m\right)\right)|\phi \left(x_{j}\left(l\right)\right)\rangle ]$ so that (22) constitutes a generalization of the Yule–Walker equations. By making l=m−n=−1,⋯,−p one may reframe (22) in matrix form as

(23) $${\bar{\kappa }}_{p}=\left[\begin{array}{c} K_{x}^{\phi }\left(-1\right)\\ \vdots \\ K_{x}^{\phi }\left(-p\right) \end{array}\right]=\left[A_{1}\;\cdots A_{p}\right]\left[\begin{array}{cccc} K_{x}^{\phi }\left(0\right) & K_{x}^{\phi }\left(-1\right) & \cdots & K_{x}^{\phi }\left(-p+1\right)\\ K_{x}^{\phi }\left(1\right) & K_{x}^{\phi }\left(0\right) & \cdots & K_{x}^{\phi }\left(-p+2\right)\\ \vdots & \ddots & \ddots & \vdots \\ K_{x}^{\phi }\left(p-1\right) & K_{x}^{\phi }\left(p-2\right) & \cdots & K_{x}^{\phi }\left(0\right) \end{array}\right]=AK_{p}\left(0\right),$$

where $K_{p}\left(0\right)$ is block Toeplitz matrix containing *p* Toeplitz blocks. Equation (23) provides $pD^{2}$ equations for the same number of unknown parameters in A.

The high model order counterpart to (17) is given by

(24) $$\Sigma_{\langle \tilde{w}\left(n\right)|}=K_{x}^{\phi }\left(0\right)-\sum_{k=1}^{p}\sum_{l=1}^{p}A_{k}K_{x}^{\phi }\left(k-l\right)A_{l}^{⊤}=K_{x}^{\phi }\left(0\right)-AK_{p}\left(0\right)A^{⊤}.$$

It is not difficult to see that the more usual Yule–Walker complete equation form becomes

(25) $$\left[I\;-A\right]\;K_{p+1}\left(0\right)=\left[\begin{array}{c} \Sigma_{\langle \tilde{w}\left(n\right)|}\\ 0 \end{array}\right].$$

There are a variety of ways for solving for the parameters. A simple one is to define a=vec(A) leading to

(26) $$\mathrm{vec}\left({\bar{\kappa }}_{p}\right)=\left(K_{p}^{⊤}\left(0\right)⊗I\right)\;a.$$

Even though one may employ least-squares solution methods to solve either (26) or (23), a Total-Least-Squares (TLS) approach [20] has proven a better solution since both members of the equations are affected by estimation inaccuracies that are better dealt with using TLS.

Likewise, (24) can be used in conjunction with generalizations of model order criteria of Akaike’s AIC type

(27) $${}_{g}\mathrm{AIC}\left(k\right)=\ln\left(\det\left(\Sigma_{\langle \tilde{w}\left(n\right)|}\right)\right)+\frac{c_{n_{s}}}{n_{s}}kD^{2},$$

where $n_{s}$ stands for the number of available time observations. In generalizing Akaike’s criterion to the multivariate case $c_{n_{s}}=2$, whereas $c_{n_{s}}=\ln\left(\ln\left(n_{s}\right)\right)$ for the Hannan–Quinn criterion, our choice in this paper.

Thus far, we have described procedures for choosing model order in the F space. In ordinary time series analysis, in addition to model order identification, one must also perform proper model diagnostics. This entails checking for residual whiteness among other things. This is usually done by checking the residual auto/crosscorrelation functions for their conformity to a white noise hypothesis.

In the present formulation because, we do not explicitly compute the F space series, we must resort to means other than computing the latter correlation functions from the residual data as usual. However, using the same ideas for computing (24), one may obtain estimates of the innovation cross-correlation in the feature space at various lags as

(28) $$\Sigma_{\langle \tilde{w}\left(n\right)|\tilde{w}\left(m\right)\rangle }=\Sigma_{\tilde{w}}\left(m-n\right)=K_{x}^{\phi }\left(m-n\right)-\sum_{k=1}^{p}\sum_{l=1}^{p}A_{k}K_{x}^{\phi }\left(m-n+k-l\right)A_{l}^{⊤},$$

by replacing $K_{x}^{\phi }\left(m-n+k-l\right)$ by their estimates and using $A_{k}$ obtained by solving (22) for m−n between a minimum −L to a +L maximum lag. The usefulness of (28) is to provide means to test model accuracy and quality as a function of ϕ choice under the best model order provided by the model order criterion.

By defining a suitable normalized estimated lagged *kernel correlation function (KCF)*

(29) $${\mathrm{KCF}}_{ij}\left(\tau \right)=\frac{K_{ij}\left(\tau \right)}{\sqrt{K_{ii}\left(0\right)K_{jj}\left(0\right)}},$$

which, given the inner product nature of kernel definition, satisfies the condition

(30) $$|{\mathrm{KCF}}_{ij}\left(\tau \right)|\leq 1,$$

as easily proved using the Cauchy–Schwarz inequality.

The notion of KFC(τ) applies not only to the original kernels but also in connection with the residual kernel values given by (28) where, for explicitness, we write it as

(31) $${\mathrm{KCF}}_{ij}^{\left(r\right)}\left(\tau \right)=\frac{\Sigma_{ij}\left(\tau \right)}{\sqrt{\Sigma_{ii}\left(0\right)\Sigma_{jj}\left(0\right)}},$$

where $\Sigma_{ij}\left(\tau \right)$ are the matrix entries in (28).

In the numerical illustrations that follow, we have assumed that ${\mathrm{KCF}}_{ij}^{\left(r\right)}\left(\tau \right)∼N\left(0,1/n_{s}\right)$ asymptotically under the white residual hypothesis

(32) $$H_{0}:{\mathrm{KCF}}_{ij}^{\left(r\right)}\left(\tau \right)=0.$$

This choice turned out to be reasonably consistent in practice. Along the same line of reasoning, other familiar tests over residuals, such as the Portmanteau test [10] were also carried out and consistently allowed verifying residual nonwhiteness.

One may say that the present theory follows closely the developments of ordinary second order moment theory with the added advantage that now nonlinear connections can be effectively captured by replacing second order moments by their respective lagged kernel estimates.

### 2.1. Estimation and Asymptotic Considerations

The essential problem then becomes that of estimating the entries of $K_{x}^{\phi }\left(n,m\right)$, entries. They can be obtained by averaging kernel values computed over the available data

(33) $$K_{ij}\left(n,m\right)=\frac{1}{n_{s}}\sum_{s}\langle \phi \left(x_{i}\left(n-s\right)\right)|\phi \left(x_{j}\left(m-s\right)\right)\rangle ,$$

for nonzero terms in the $s\in [1,n_{s}]$ range.

Under these conditions, for an appropriately defined kernel function, the feature space becomes linearized and, following [21], it is fair to assume that the estimated vector stacked representation of the model coefficient matrices

(34) $$a=\mathrm{vec}(\left[A_{1}\cdots A_{p}\right])$$

is asymptotically Gaussian, i.e.,

(35) $$\sqrt{n_{s}}\left(\hat{a}-a\right)∼N\left(0,\Gamma^{-1}⊗\Sigma_{\langle \tilde{w}\left(n\right)|}\right),$$

where $\Sigma_{\langle \tilde{w}\left(n\right)|}$ is the feature space residual matrix given by (28) and where

(36) $$\Gamma =E[y_{L}y_{R}^{⊤}]$$

for the ‘bra’-vector

(37) $$y_{L}^{⊤}={[\langle \phi \left(x\left(n\right)\right)|,\cdots ,\langle \phi \left(x\left(n-p+1\right)\right)|]}^{⊤}$$

and the ‘ket’-vector

(38) $$y_{R}^{⊤}={[|\phi \left(x\left(n\right)\right)\rangle ,\cdots ,|\phi \left(x\left(n-p+1\right)\right)\rangle ]}^{⊤},$$

which are used to construct the kernel scalar products. It is immediate to note that (36) is a Toeplitz matrix composed of suitably displaced $K_{x}^{\phi }\left(\cdot \right)$ blocks.

An immediate consequence of (35) is that one may test for model coefficient nullity and thereby provide a kernel Granger Causality test. This is equivalent to testing for $a_{ij}\left(k\right)=0$ so that the statistic

(39) $${}_{g}\lambda_{W}={\hat{a}}^{⊤}C^{⊤}{\left[C\left(\Gamma^{-1}⊗\Sigma_{\langle \tilde{w}\left(n\right)|}\right)C^{⊤}\right]}^{-1}C\hat{a},$$

where C is a contrast matrix (or structure selection matrix) so that the null hypothesis becomes

(40) $$H_{0}:Ca=0.$$

Hence, under (35),

(41) $${}_{g}\lambda_{W}\overset{d}{\to }\chi_{\nu }^{2},$$

where ν=rank(C) corresponds to the number of the explicitly imposed constraints on $a_{ij}\left(k\right)$.

#### Data Workflow

Given $x_{i}\left(n\right)$, analysis proceeds by

1. Computing the kernel values (33) to obtain the kernel Yule–Walker equations (25) or equivalently (26) for a given value of *p* (starting from p=1);
2. After solving the latter for the parameters in a via Total-Least-Squares (TLS), one computes (18) wherefrom the generalized model order choice criterion (27) can be computed;
3. With the help of the computed (33) values, one can obtain the residual ${\mathrm{KCF}}_{ij}^{\left(r\right)}\left(\tau \right)$ functions in (31) which can be used to check model adequacy via (32). Additionally, Portmanteau tests may be also used;
4. If ${\mathrm{KCF}}_{ij}^{\left(r\right)}\left(\tau \right)$ analysis does not suggest feature space model residual whiteness, *p* is increased by 1, and the procedure from step 1 is repeated until feature space model residual whiteness is obtained and ${}_{g}\mathrm{AIC}\left(k\right)$ attains its first local minimum meaning that the ideal model order has been reached;
5. Once the best model is attained, one employs the (39) to infer connectivity.

These steps closely mirror those of ordinary time series model fitting and analysis.

## 3. Numerical Illustrations

The following examples consist of nonlinearly coupled systems that are simulated with the help of zero mean unit variance normal uncorrelated innovations $w_{i}\left(n\right)$. All simulations (10,000 realizations each) were preceded by an initial a burn-in period of 10,000 data points to avoid transient phenomena. Estimation results are examined as a function of $n_{s}=\left\{32,64,128,256,512,1024,2048\right\}$ with α=1% significance.

For brevity, Example 1 is carried out in full detail, whereas approach performance for the other ones is gauged mostly through the computation of observed detection rates except for Examples 4 and 5 which also portray model order choice criterion behaviour.

Simulation results are displayed in terms of how true and false detection rates depend on realization length $n_{s}$.

### 3.1. Example 1

Consider the simplest possible system whose connectivity cannot be captured by linear methods [17] as there is a unidirectional quadratic coupling from $x_{2}\left(n\right)$ to $x_{1}\left(n\right)$

(42) $$\left\{\begin{array}{cc} x_{1}\left(n\right) & =ax_{1}\left(n-1\right)+cx_{2}^{2}\left(n-1\right)+w_{1}\left(n\right),\\ x_{2}\left(n\right) & =bx_{2}\left(n-1\right)+w_{2}\left(n\right), \end{array}\right.$$

with a=0.2, b=0.6 and c=0.7.

An interesting aspect of this simple system is the possibility of easily relating its coefficients *a*, *b* and *c* to those in (14) that describe its F space evolution. This may be carried out explicitly after substituting (42) into the computed kernels of Equation (13). After a little algebra, this leads to

(43) $$\left[\left[\begin{array}{cc} a & 0\\ 0 & b \end{array}\right]-\left[\begin{array}{cc} \alpha_{11} & \alpha_{12}\\ \alpha_{21} & \alpha_{22} \end{array}\right]\right]=\left[\begin{array}{cc} c & 0\\ 0 & 0 \end{array}\right]\left[\begin{array}{cc} \theta_{11} & \theta_{12}\\ 0 & 0 \end{array}\right],$$

where $\theta_{11}$ and $\theta_{12}$ depend on the computed kernel values. From (43), it immediately follows for example that $b=\alpha_{22}$ and more importantly that $\alpha_{21}=0$ as expected. Vindication of the observation of these theoretically determined values also gives the means for testing estimation accuracy.

For illustrations’ sake, we write the kernel Yule–Walker Equations (22) with their respective solutions ($n_{s}=512$) for one given (typical) realization

(44) $$\left[\begin{array}{cc} 210.7583 & 23.5416\\ 23.5416 & 8.6450 \end{array}\right]A^{\left(2\right)}=\left[\begin{array}{cc} 125.7501 & 37.7803\\ 17.7389 & 5.3788 \end{array}\right]\to A^{\left(2\right)}=\left[\begin{array}{cc} 0.1559 & 3.9456\\ 0.0211 & 0.5648 \end{array}\right],$$

for the quadratic kernels ($\kappa \left(x,y\right)={\left(xy\right)}^{2}$) and

(45) $$10^{5}\times \left[\begin{array}{cc} 8.0302 & 0.0868\\ 0.0868 & 0.0052 \end{array}\right]A^{\left(4\right)}=10^{5}\times \left[\begin{array}{cc} 4.1597 & 0.1755\\ 0.0594 & 0.0025 \end{array}\right]\to A^{\left(4\right)}=\left[\begin{array}{cc} 0.1843 & 30.8922\\ 0.0027 & 0.4386 \end{array}\right],$$

for the quartic kernels ($\kappa \left(x,y\right)={\left(xy\right)}^{4}$). Superscripts point to kernel order. One may readily notice approximate compliance to the expected $\alpha_{ij}$ coefficients.

**Figure 1:**
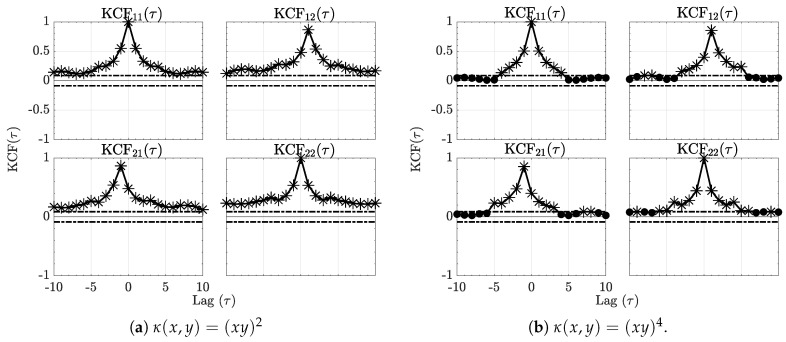
The sequence kernel correlation functions (KCF(τ)) respectively for the quadratic and quartic kernels are contained in Figure 1a,b for Example 1. Horizontal dashed lines represent 95% significance threshold interval out of which the null hypothesis $H_{0}$ of no correlation is rejected. Asterisks (*) further stress signficant values.

Further appreciation of this example may be obtained via a plot of the normalized estimated KCF(τ) (29) shown in Figure 1.

**Figure 2:**
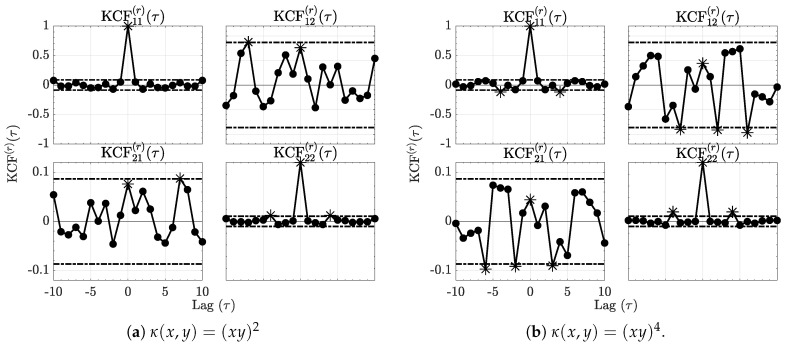
The residue kernel correlation functions (KCF${}^{\left(r\right)}\left(\tau \right)$) respectively for the quadratic and quartic kernels are shown in Figure 2a,b for Example 1. Comparing them to Figure 1, it is clear that the kernel correlations are reduced after modelling as it is now impossible to reject KCF${}^{\left(r\right)}\left(\tau \right)$ nullity at 95% as no more than 5% of the values lie outside the dashed interval around zero. Asterisks (*) further stress significant values.

The residual normalized kernel sequences (31) computed using (28) are depicted in Figure 2 for each kernel and show effective decrease below the null hypothesis decision threshold line vindicating adequate modelling.

Moreover, for this realization, one may show that the Hannan–Quinn Information Criterion (27) points to the correct order of p=1. In addition, Portmanteau tests do not reject whiteness in the F space for either kernel further confirming successful modelling in both cases.

**Figure 3:**
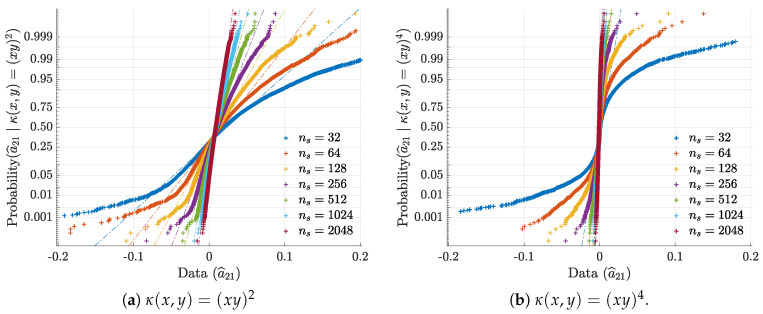
Ensemble normal probability plots for ${\hat{a}}_{21}$, respectively for 3a quadratic and 3b quartic kernels, illustrate and confirm asymptotic normality.

**Figure 4:**
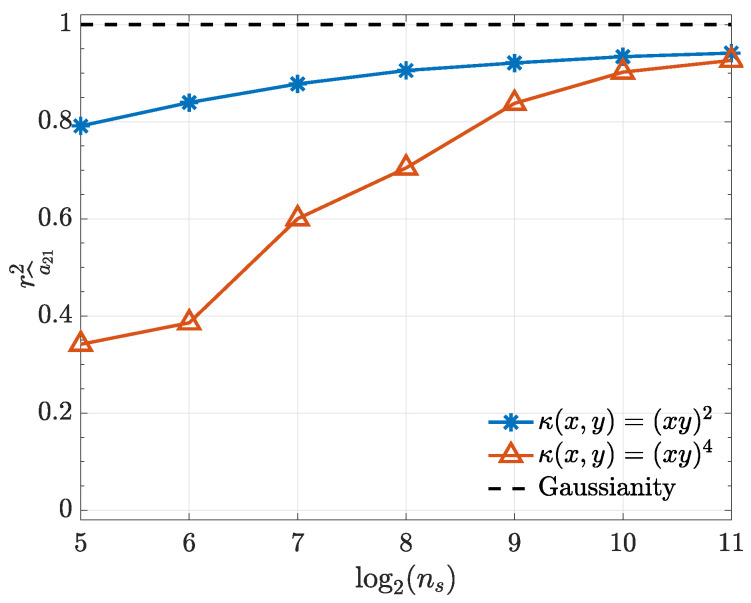
*Filliben* squared-correlation coefficient convergence to Gaussianity as a function of $n_{s}$ for both kernels used in Example 1.

To illustrate and confirm the Gaussian asymptotic behaviour discussed in Section 2.1, normal probability plots for ${\hat{a}}_{21}$ are presented in Figure 3. Further objective quantification of the convergence speed towards normality is provided by the evolution towards 1 of the *Filliben* squared-correlation coefficient [22,23,24] as a function of $n_{s}$ (Figure 4).

**Figure 5:**
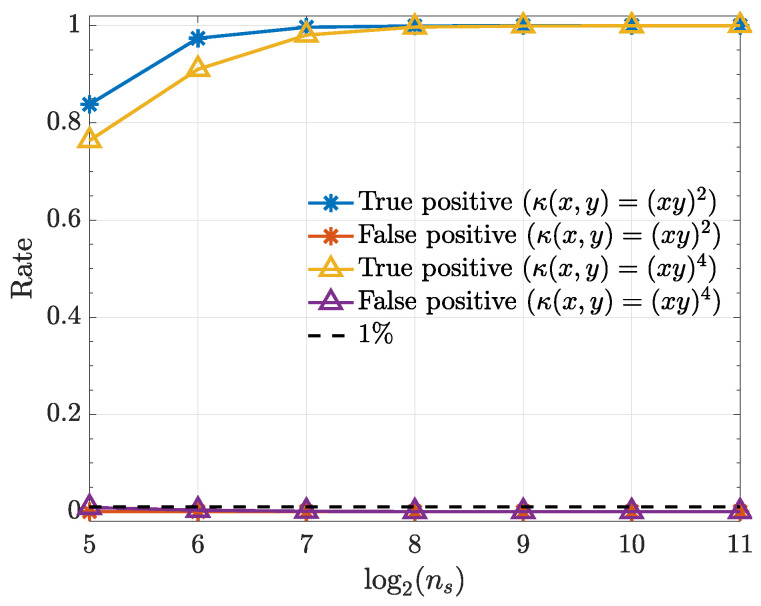
True positive and false positive rates from the kernelized Granger causality test for various samples sizes ($n_{s}$) for α=1%. Note that the false-positive-rates for both kernels overlap.

Convergence to normality justifies using (39) to test for null connectivity hypotheses. Test perfomance is depicted in Figure 5.

### 3.2. Example 2

Consider $x_{1}\left(n\right)$, a highly resonant (R=0.99) linear oscillator (at a normalized frequency of f=0.1) to be unidirectionally coupled to a low pass system $x_{2}\left(n\right)$ through a delayed squared term

(46) $$\left\{\begin{array}{cc} x_{1}\left(n\right) & =2R\cos\left(2\pi f\right)x_{1}\left(n-1\right)-R^{2}x_{1}\left(n-2\right)+w_{1}\left(n\right),\\ x_{2}\left(n\right) & =-0.9x_{2}\left(n-1\right)+cx_{1}^{2}\left(n-1\right)+w_{2}\left(n\right), \end{array}\right.$$

where c=0.1 [17].

**Figure 6:**
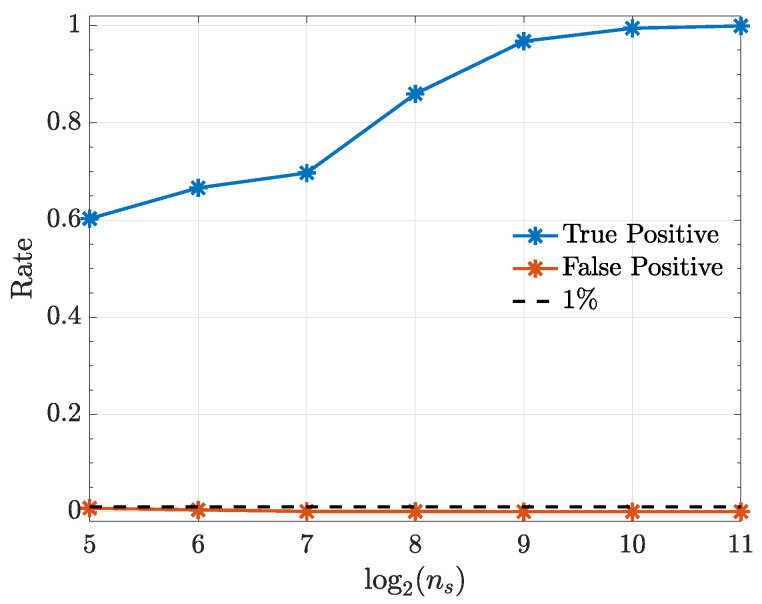
True positive ($x_{1}\to x_{2}$) and false positive rates ($x_{2}\to x_{1}$) from the kernelized Granger causality test under a quadratic kernel as a function $n_{s}$ in Example 2.

This system was already investigated elsewhere [17,25,26] under a different estimation algorithm and with fewer Monte Carlo replications. The null hypothesis connectivity results are presented in Figure 6 showing adequate asymptotic decision success. A quadratic kernel was used in all cases.

### 3.3. Example 3

The present example comes from a model in [27],

(47) $$\left\{\begin{array}{cc} x_{1}\left(n\right) & =3.4x_{1}\left(n-1\right){[1-x_{1}^{2}\left(n-1\right)]e}^{-x_{1}^{2}\left(n-1\right)}+w_{1}\left(n\right),\\ x_{2}\left(n\right) & =3.4x_{2}\left(n-1\right){[1-x_{2}^{2}\left(n-1\right)]e}^{-x_{2}^{2}\left(n-1\right)}+c_{1}x_{1}^{2}\left(n-1\right)+w_{2}\left(n\right),\\ x_{3}\left(n\right) & =3.4x_{3}\left(n-1\right){[1-x_{3}^{2}\left(n-1\right)]e}^{-x_{3}^{2}\left(n-1\right)}+c_{2}x_{2}^{4}\left(n-1\right)+w_{3}\left(n\right). \end{array}\right.$$

This choice was dictated by the nonlinear wideband character of its signals. The values $c_{1}=0.7$ and $c_{2}=0.9$ were adopted.

**Figure 7:**
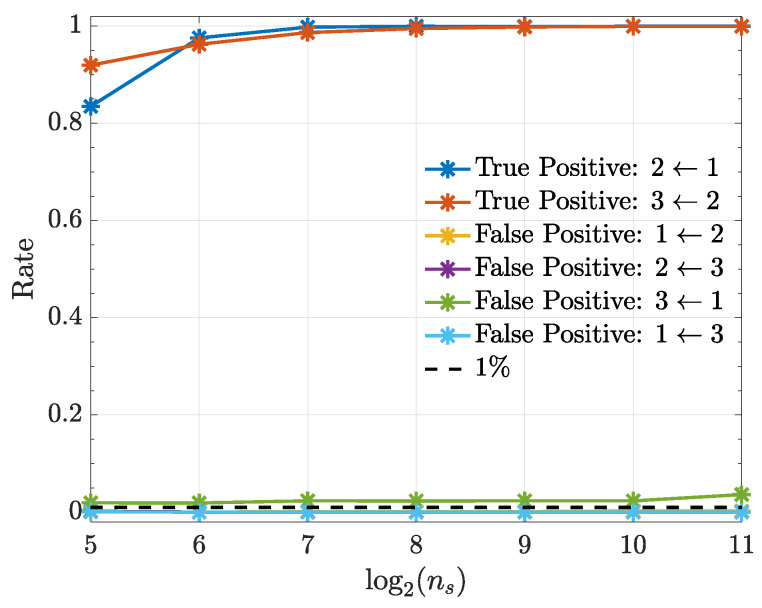
True positive and false positive rates (Example 3) from the kernelized Granger causality test using a quadratic kernel as a function of $n_{s}$. Note that the false-positive-rate for the connections 1←2, 2←3 and 1←3 overlap over the investigated $n_{s}$ range.

Figure 7 shows that connection detectability improves as signal duration $n_{s}$ increases except for the nonexisting $x_{3}\left(n\right)\leftarrow x_{1}\left(n\right)$ connection whose performance stays more or less constant with a false positive rate slightly above α=1%. All computations used quadratic kernels.

### 3.4. Example 4

For this numerical illustration, consider the model presented in [28]

(48) $$\left\{\begin{array}{cc} x_{1}\left(n\right) & =3.4x_{1}\left(n-1\right){[1-x_{1}^{2}\left(n-1\right)]e}^{-x_{1}^{2}\left(n-1\right)}+0.8x_{1}\left(n-2\right)+w_{1}\left(n\right),\\ x_{2}\left(n\right) & =3.4x_{2}\left(n-1\right){[1-x_{2}^{2}\left(n-1\right)]e}^{-x_{2}^{2}\left(n-1\right)}+0.5x_{2}\left(n-2\right)+cx_{1}^{2}\left(n-2\right)+w_{2}\left(n\right). \end{array}\right.$$

System (48) produces nonlinear wideband signals with a quadratic (1→2) coupling factor whose intensity is given by *c* taken here as 0.5.

**Figure 8:**
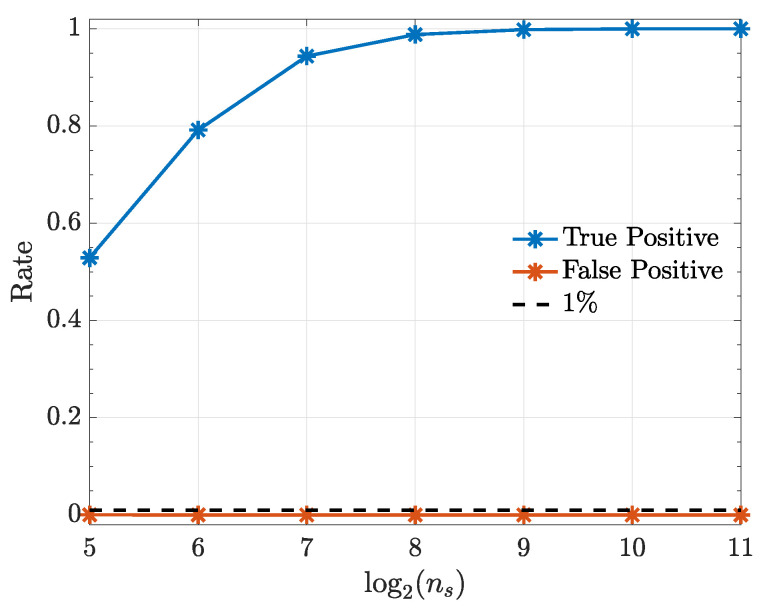
True positive and false positive rates from the kernelized Granger causality test under a quadratic kernel as function of record length $n_{s}$ in Example 4.

**Figure 9:**
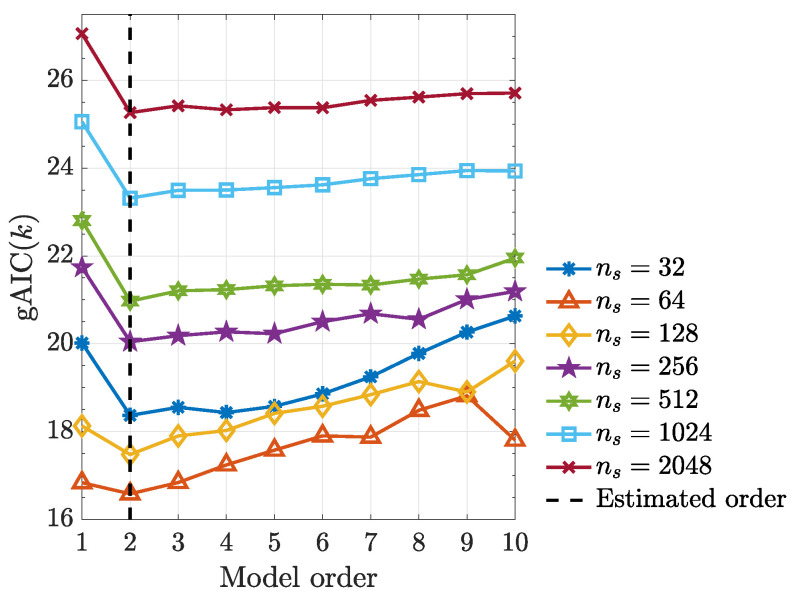
Generalized Hannan–Quinn criterion (${}_{g}\mathrm{AIC}\left(k\right)$) with $c_{n_{s}}=\ln\left(\ln\left(n_{s}\right)\right)$ as a function of model order for various observed record lengths $n_{s}$ using a typical realization from (48).

It is worth noting that, kernelized Granger causality true positive rate improves as sample size ($n_{s}$) increases (Figure 8) and using the generalized Hannan–Quinn criterion, the order of kernelized autoregressive vector models identified for a typical realization was correctly identified and equals 2 as expected (see Figure 9).

### 3.5. Example 5

As a last numerical illustration, consider data generated by

(49) $$\left\{\begin{array}{cc} x_{1}\left(n\right) & =3.4x_{1}\left(n-3\right){[1-x_{1}^{2}\left(n-3\right)]e}^{-x_{1}^{2}\left(n-3\right)}+0.4x_{1}\left(n-4\right)+w_{1}\left(n\right),\\ x_{2}\left(n\right) & =3.4x_{2}\left(n-1\right){[1-x_{2}^{2}\left(n-1\right)]e}^{-x_{2}^{2}\left(n-1\right)}+c_{1}x_{1}^{2}\left(n-2\right)+w_{2}\left(n\right),\\ x_{3}\left(n\right) & =3.4x_{3}\left(n-2\right){[1-x_{3}^{2}\left(n-2\right)]e}^{-x_{3}^{2}\left(n-2\right)}+c_{2}x_{2}^{2}\left(n-3\right)+w_{3}\left(n\right), \end{array}\right.$$

with $c_{1}=0.9$ and $c_{2}=0.4$.

**Figure 10:**
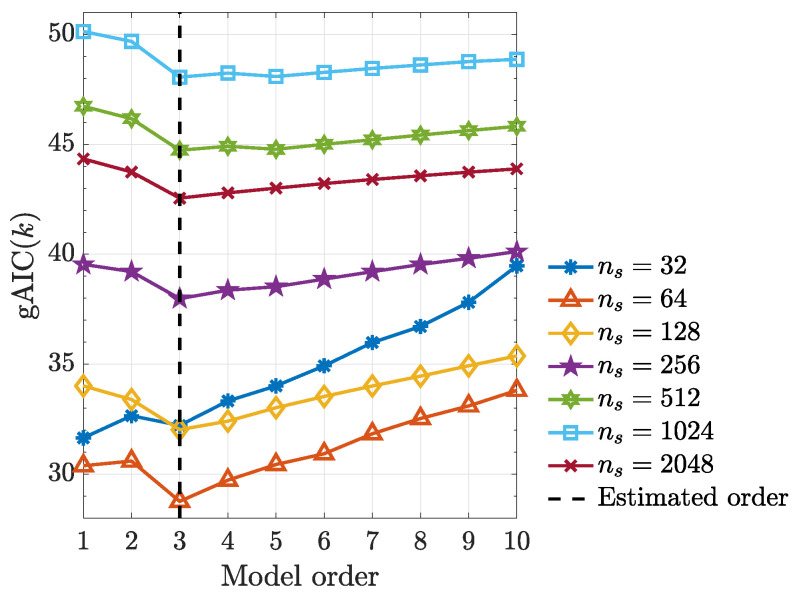
Generalized Hannan–Quinn criterion (${}_{g}\mathrm{AIC}\left(k\right)$) as a function of model order for the various data lengths $n_{s}$ from a typical realization from (49).

**Figure 11:**
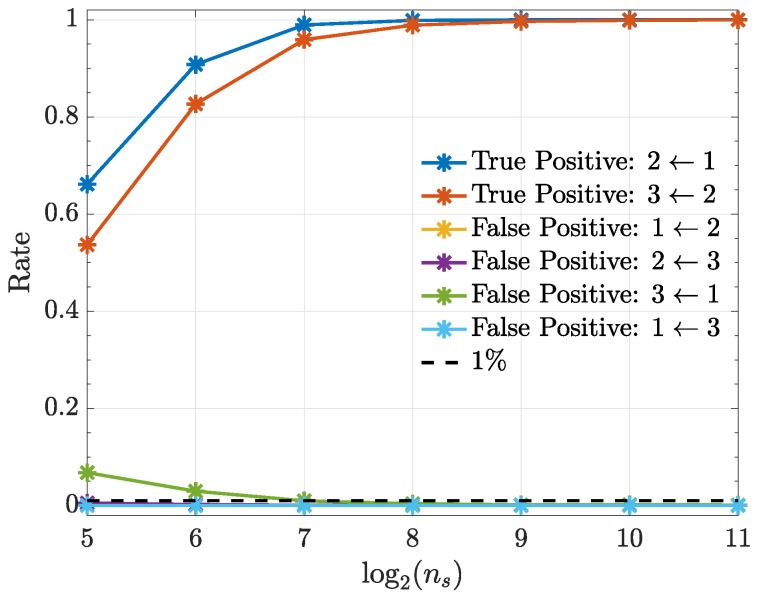
Observed true positive and false positive rates from the kernelized Granger causality test under a quadratic kernel for various record lengths $n_{s}$ in Example 5. Note that the false-positive-rate for the connections 2←3, 3←1 and 1←3 overlap over the $n_{s}$ range, except for 1←2, which, however, attains the same level as the others after $n_{s}=128$.

Under the quadratic kernel and employing kernelized Hannan–Quinn information criterion (27) (see Figure 10), one can see that the estimated model order is p=3 as expected judging from the $x_{2}^{2}\left(n-3\right)$ term in (49). In addition, kernelized Granger causality detectability improves with record length $n_{s}$ increase (Figure 11).

## 4. Conclusions and Future Work

After a brief theoretical presentation (Section 2), we have shown that canonical model fitting procedures that involve (a) model specification with order determination and (b) explicit model diagnostic testing can be successfully carried out in the feature space F to detect connectivity via reproducing kernels. In dealing with Granger causality detection using kernels as in [29,30], this stands in sharp contrast as the latter depend on solving the *reconstruction/pre-image* problem to provide prediction error estimates in the original data space X. In fact, part of the challenge in pre-image determination lies in its frequently associated numerical ill-condition [31].

The key result behind doing model diagnostics and inference in F is (28) by realizing that kernel quantities may be normalized much as correlation coefficients. It should be noted that (28) holds even in the case of (nonkernel) linear modelling by replacing the K matrices by auto/crosscorrelation matrices, something that, in practice, is never adopted in classical linear time series modelling because the necessary auto/crosscorrelations are more efficiently computed from model residuals that are easy to obtain as no pre-imaging problem is involved there.

Thus, what importantly sets the present approach apart from previous work is the lack of need for returning to the original input space X to gauge model quality as the *reconstruction/pre-image problem* can be fully circumvented bypassing unnecessary uncertainties.

As such, we showed that, because model adequacy testing can be performed *directly* in the feature space F, directional Granger type connectivity can be detected for a variety of multivariate nonlinear coupling scenarios, thereby totally dispensing with the need for detailed ‘a priori’ model knowledge.

We observed that successful connectivity detection is achievable at the expense of a relatively short time series. A systematic comparison with other approaches [4,5,32,33,34,35] is planned for future work, but, at least for the cases we tested so far, savings of at least one order magnitude in record lengths are feasible.

One of the basic tenants of the present work is that model coefficients in the feature space are asymptotically normal, something whose consistency was successfully illustrated though the need for a more formal proof remains, especially in connection to explicit kernel estimates under the total-least-squares solution to (23). Our choice of TLS was dictated by its apparent superiority when compared to the ‘kernel trick’ [32] whose multivariate version we employed in [26,36,37].

In this context, it is important to note that, contrary to other methods that require time-consuming resampling procedures for adequate inference, the present approach relies on asymptotic statistics and is thus less susceptible to eventual problems derived from data shuffling.

One of the advantages of the present development is that the procedure allows for determining how far in the past to look via the model order criteria we employed (27).

Even though order estimation and model testing were successful upon borrowing from the usual linear modelling practices, further systematic examination is still needed and is under way.

One may rightfully argue that the kernels we chose for illustrating the present work are equivalent to modelling the original time series after the application of a suitable ϕ(·) transformation to the data and that they look for the causality evidence present in higher order momenta. This, in fact, explains why quadratic kernels converge much faster than quartic ones in Example 1. The merit of framing the time series transformation discussion for connectivity detection in terms of kernels produces a simple workflow and paves the way to developing future data-driven criteria towards optimum data transformation choice for a given problem. Other kernel choices are being investigated.

The signal model used in the present development does not contemplate additive measurement noise whose impact on connectivity detection we also leave for future examination.

One thing the present type of analysis cannot do is expose details of how the nonlinearity takes place. For example, coupling may be quadratic or involve higher exponential powers or some other function. What the present approach can do, however, is to expose existing connections, so that modelling efforts can be concentrated on them, thereby avoiding modelling parameter waste on non relevant links.

Finally, the present systematic empirical investigation sets the proposal of using feature space-frequency domain descriptions of connectivity like *kernel partial directed coherence* [26,36], and *kernel directed transfer function* [37] on sound footing, especially with respect to their asymptotic connectivity behaviour.
